# Communication and humanization of care: Effects over burnout on nurses

**DOI:** 10.1371/journal.pone.0251936

**Published:** 2021-06-10

**Authors:** María del Mar Molero Jurado, Iván Herrera-Peco, María del Carmen Pérez-Fuentes, Nieves Fátima Oropesa Ruiz, África Martos Martínez, Diego Ayuso-Murillo, Jose Jesús Gázquez Linares

**Affiliations:** 1 Department of Psychology, Faculty of Psychology, University of Almería, Almería, Spain; 2 Nursing Department, Faculty of Health Sciences, Alfonso X El Sabio University, Madrid, Spain; 3 Alfonso X El Sabio Foundation, Madrid, Spain; 4 Consejo General de Enfermería, Madrid, Spain; 5 Department of Psychology, Universidad Autónoma de Chile, Providencia, Chile; Murcia University, SPAIN

## Abstract

**Background:**

Healthcare professionals may have certain psychological characteristics which contribute to increasing the quality of their professional performance.

**Objective:**

Study the effect that humanization of care and communication have on the burnout syndrome in nursing personal.

**Methods:**

The sample included a total of 330 Spanish nurses. Analytical instruments used were the Health Professional’s Humanization Scale (HUMAS), Communication Styles Inventory Revised (CSI-R) and Brief Burnout Questionnaire Revised (CBB-R).

**Results:**

Two broad nursing profiles could be differentiated by their level of humanization (those with scores over the mean and those with scores below it in optimistic disposition, openness to sociability, emotional understanding, self-efficacy, and affection), where the largest group had the high scores. A communication repertoire based on verbal aggressiveness impacted indirectly on the effect of humanization on burnout, mainly in the personal impact component. We observed the relation of humanization profiles in nursing staff with the job dissatisfaction and burnout components. Besides that, some communication styles, verbal aggressiveness and questioningness, have an indirect effect on the relationship between humanization profiles and job dissatisfaction.

**Conclusions:**

The results on the relationship between communication styles and burnout, and the mediator effect of communication styles on the relationship between humanization of care and burnout in nursing personnel are discussed.

## Introduction

Healthcare professionals may have certain psychological characteristics which contribute to increasing the quality of their performance. In nurses, as an example of professionals where contact with patients is direct and continuous, certain personal resources have been observed (self-esteem, emotional intelligence, self-efficacy, openness to communication, social skills and empathy) which may enable them to fight stress and psychophysical exhaustion better [1–4]. At the present time, in the field of health and in care professions, the importance of patient-centered care is being recovered to improve care quality [5, 6]. Starting out from this theoretical framework, we understand that for care to be humanized, the health professional must have the following characteristics: optimistic disposition, emotional understanding, sociability, self-efficacy and affection [7]. Disposition to optimism, which refers to positive expectations for the future; sociability, which refers to the ability to relate to others through assertiveness and empathy; emotional understanding, which involves understanding and interpreting the feelings of other persons correctly; self-efficacy, which means trusting in one’s own actions to achieve the expected results in potentially stressful situations; and affection, which consists of empathizing emotionally with the affective state of the other person without fusion between the feelings of oneself and others. From this perspective, humanization contributes to the integral development of the human being through a global approach to healthcare, where the patients become the center of the system and take on an active role, along with the healthcare professional, in caring for their own health [7]. The idea is to ensure professional commitment to improving care quality [2, 8], considering that the main goal of healthcare professionals must be to improve the patient’s overall health [9], with intervention incorporating agents involved in health and wellbeing [10–13].

Furthermore, patients must be convinced that they are receiving adequate care [14, 15], and assertive, effective communication has been shown to be one of the main vehicles for achieving this [16–19]. Thus, in a nursing population, verbal aggressiveness has been significantly related positively to agreeableness, conscientiousness and neuroticism, and although the mood dimension of emotional intelligence moderated this relationship, it was only relevant for those persons with high scores in neuroticism [20]. In other studies, emotional intelligence has been positively related to communication skills [21]. It may be deduced from this that integral humanized patient care requires effective communication, especially in highly psychosocially stressful situations (such as in terminal illness or emergencies), where complex communication takes place between professionals, patients and family members [10, 22–25]. The literature shows that when communication is inappropriate, it could generate adverse situations both for patients and healthcare professionals. With respect to the first ones, an inappropriate communication from healthcare professionals could cause stress and non-adherence to the treatment [26]. However, with regard to an inappropriate communication between healthcare professionals, it could generate confusion and loss of confidence among team members even in addition to stress, job dissatisfaction and emotional exhaustion [27–29]. Training in communication skills is an effective way of reducing burnout among nurses and improving job satisfaction [30, 31].

To contribute to improving health qualitatively and significantly, we should start with contributions from positive psychology, which focuses on developing personal traits contrary to burnout [3, 32]. Some authors suggest that certain personal resources, such as self-efficacy, which in turn are related to self-esteem and integration in the setting [1], improve work commitment. Related to self-efficacy, strong prosocial motivation in healthcare professionals has been associated with higher levels of burnout, because of the difficulty such professionals experience in healthy distancing from the tensions derived from the care relationship, and they are therefore affected by that relationship. On the contrary, high levels of intrinsic motivation, even extrinsic, has been associated with less burnout [33]. Care quality has also been associated with active listening or the capacity for cognitive empathy with the thoughts, feelings and intentions of others [3]. In this line, it has been shown that in clinical practice, cognitive empathy improves communication with the patient and their adherence to treatment [34].

After analyzing the empirical results of the most relevant studies related to the subject of this study, the main objectives were: (1) Determine the exploratory value of communication styles and burnout in humanization in a sample of nursing professionals. (2) Explore the mediating role of influence of communication styles on the relationship between humanization profile and the burnout components. With regard to these objectives, and starting out from previous empirical evidence, the following research hypotheses were: (I) determine the explanatory value of the dimensions associated with humanization and burnout in communication styles. (II) It was expected for certain components of Humanization and burnout to have more explanatory weight in communication styles. (III) It was expected a negative mediating role for burnout in communication styles. (IV) It was expected an indirect influence of communication styles on the relationship between humanization profiles and the burnout components.

## Methods

### Participants

Our original sample consisted of 338 registered nurses working at Spanish hospitals, who participated voluntarily in this study. Inclusion criteria was: Nursing professionals in active employment status at the time of data collection. Participants filling out the questionnaire to whom those incomplete or with random answers, found by control questions distributed at random throughout the battery of questionnaires, were eliminated from the study. This system of answer control is based on questions with a single answer which is obviously correct, such as, “*Right now I am answering a survey*.*”* Eight participants were eliminated due to errors in answers to control questions, leaving a final sample of 330 registered nurses, with a mean age of 32.30 (*SD* = 7.54; range 22–56). Regarding gender, 83.9% (*n* = 277) were women and 16.1% (*n* = 53) were men, with mean ages of 32.62 (SD = 7.92) and 30.62 (SD = 4.90), respectively.

### Procedure

This cross-sectional study was done with snowball sampling carried out on social networks and instant messaging. The participants filled out the tests individually in a time estimated at 5–10 minutes.

All participants responded voluntarily and gave their written informed consent prior to filling out the questionnaire, after being informed of the objectives of the research and the anonymous nature of their answers. The data were collected and processed respecting all of the rights and guarantees as provided for in EU Regulation 2016/679 and Organic Law 3/2018 of December 5th on Protection of Personal Information and guarantee of digital rights.

The questionnaire was implemented as a CAWI (Computer Aided Web Interviewing) interview, in which the participants expressly gave their consent by marking a box for the purpose before going to the questionnaire screen.

### Instruments

The sociodemographic data were collected in ad hoc questionnaire, which included items like age or sex among others.

*Escala de Humanización en el Profesional Sanitario—Health Professsional´s Humanization Scale* (HUMAS [7]). Humanization is defined based on an Optimistic disposition, which refers to positive expectations about future events; Sociability, or preference for seeking the company of others with whom assertive and empathic relations are maintained; Emotional understanding, which is understanding, identifying and interpreting rationally the feelings and emotions experienced by others; Self-efficacy or trusting your own actions to manage complicated, stressful situations successfully; Affection or processing emotional information, empathizing with the affective state of the other person. These five dimensions are evaluated in 19 items (five for affection and self-efficacy and three for the rest), which are scored on a Likert-type scale from 1 (never) to 5 (always). The reliability analysis showed high consistency in the dimensions above.

The *Communication Style Inventory Revised* (CSI-R [35]) is a revised version of the original questionnaire designed by Vries et al. [36]. It measures the predominance of certain types of behavior in communication in 96 items rated on a Likert-type scale from 1 (totally disagree) to 5 (totally agree). It provides information on six dimensions: Verbal aggressiveness or communication that is hostile, cold, authoritarian and disrespectful with the other person; Impression manipulativeness, making use of happiness, but at the same time, hiding information; Questioningness, which searches for the detail and corroborates one’s own point of view; Expressiveness, in which conversation is relaxed, humorous and controlled; Emotionality or communication loaded with sentimentalism, tension, worry and defensive attitudes; Preciseness or the way that one structure dialogue making it concise and emphasizing what is important.

*Brief Burnout Questionnaire Revised* (CBB-R [37]). This is a revised version for healthcare personnel based on the Brief Burnout Questionnaire. It measures the burnout syndrome in 15 items rated on a Likert-type scale from 1 (hardly ever) to 5 (most of the time). It provides information on four components of burnout: Personal impact, referring to the effect of burnout in different areas of the worker’s life; Job dissatisfaction, referring to the balance between expectations and achievements in the workplace and enjoying one’s work; Social climate, the extent to which stressful interpersonal situations occur in the workplace; and Quitting motivation, due to lack of personal growth and promotion in the work environment.

### Data analysis

To identify the relationships between variables in the study, descriptive and correlational analyses were carried out, including: Humanization (Optimistic disposition, Sociability, Emotional understanding, Self-efficacy and Affection), Communication (Verbal aggressiveness, Impression Manipulativeness, Questioningness, Expressiveness, Emotionality and Preciseness), and Burnout (Personal impact, Job dissatisfaction, Social climate and Quitting motivation).

To examine the reliability of the instruments used for data collection, the following procedure was used to estimate the internal consistency of the scores: 1) First, an exploratory factor analysis was carried out on the polychoric correlation matrix, using the FACTOR software [38]. The data are computed under a criterion of parametric analysis and Promin rotation. 2) To calculate the alpha ordinal coefficient, the Excel spreadsheet developed by Domínguez-Lara [39] was used. This provides data on the alpha ordinal coefficient, based on the data of the polychoric correlation analysis and, therefore, is more suitable for calculating the reliability of scales with ordinal response or based on a Likert scale [40, 41]. Then the different Humanization component profiles were identified. To do this, a two-stage cluster analysis with automatic classification was performed. This procedure determines the "optimal" number of clusters automatically using the criteria specified for the cluster, in this case, Log-Likelihood. A Student’s *t* test for independent samples was applied to find out whether there were any statistically significant differences between the groups in Communication and Burnout, using the Cohen’s *d* to quantify the effect size.

In order to identify the communication styles with the most weight in the prediction of each of the Burnout dimensions, a stepwise multiple linear regression analysis was performed. Later, based on these results, simple mediation models were computed for the Burnout components, including the communication parameters with significant weight in the regression equation as potential mediators, using the PROCESS macro for SPSS developed by Hayes [42]. For the analysis of the indirect effects, bootstrapping with 5000 bootstraps was used.

### Ethical issues/statement

Prior to collecting data, we assured the participants that the treatment of data in the study would comply with applicable standards of data security, confidentiality and ethics. The study was approved by the Bioethics Committee (Ref: UALBIO2019/30) of the University of Almería (Spain). The questionnaire was applied on a web platform which enabled subjects to complete them online. A series of control questions were included to monitor for random or incongruent responses, which were removed from the study.

## Results

### Humanization, communication and burnout: Descriptive and correlation analyses

The reliability analysis for Health Professsional´s Humanization Scale (HUMAS) showed high consistency in the dimensions. Specifically, the ordinal alpha coefficients were: .98 for Optimistic disposition, .92 for Sociability, .92 for Emotional understanding, .90 for Self-efficacy, and .91 for Affection.

For the Communication Style Inventory Revised (CSI-R) the following ordinal alpha values were obtained for each of the dimensions: Verbal aggressiveness (0.88), Impression manipulativeness (.91), Questioningness (.81), Expressiveness (.78), Emotionality (.71), and Preciseness (.81).

Finally, for burnout components (Brief Burnout Questionnaire Revised) the following ordinal alpha values were obtained: Personal impact (.86), Job dissatisfaction (.79), Social climate (.75), and Quitting motivation (.64).

As observed in the correlation matrix (Table 1), there are significant relationships between the Humanizations dimensions and communication styles. The first Humanization component, Optimistic disposition, showed negative correlations with Verbal aggressiveness and Emotional communication, but was positively correlated with Preciseness in communication. Sociability was negatively correlated with aggressive communication styles, Impression manipulativeness and Questioningness, and positively with Preciseness. Emotional understanding was also related positively with preciseness, and had a negative relationship with Verbal aggressiveness. Self-efficacy also correlated negatively with Verbal aggressiveness, in addition to Emotionality, and positively with Preciseness. Finally, the Affection factor showed negative correlations with all the communication styles except Preciseness, with which it had a significant relationship.

**Table 1:** Humanization, communication and burnout. Correlations and descriptive statistics.

	1	2	3	4	5	6	7	8	9	10	11	12	13	14	15
1. OD	–														
2. SO	.49***	–													
3. EU	.42***	.31***	–												
4. SE	.57***	.53***	.52***	–											
5. AF	.11*	.14**	-.06	.06	–										
6. VA	-.12*	-.23***	-.10*	-.17**	-.42***	–									
7. IM	-.07	-.17**	.01	-.10	-.31***	.55***	–								
8. QU	-.02	-.13*	-.07	-.02	-.40***	.61***	.48***	–							
9. EX	.09	-.02	.05	.10	-.17**	.42***	.34***	.50***	–						
10. EM	-.12*	-.04	-.05	-.12*	-.42***	.62***	.45***	.57***	.42***	–					
11. PR	.21***	.16**	.19***	.33***	.01	.14**	.14*	.23***	.38***	.15**	–				
12. PI	-.32***	-.20***	-.22***	-.31***	-.39***	.36***	.22***	.28***	.09	.25***	-.02	–			
13. JD	-.17**	-.16**	-.05	-.16**	-.25***	.32***	.22***	.30***	.21***	.24***	.09	.56***	–		
14. SC	.37***	.36***	.31***	.38***	.21***	-.29***	-.08	-.18**	.03	-.12*	.20***	-.50***	-.26***	–	
15. QM	-.12	-.16*	-.03	-.07	-.31***	.37***	.14*	.40***	.24***	.25***	.03	.42***	.46***	-.30***	–
*M*	4.35	4.61	3.85	4.18	3.63	1.96	1.75	2.30	2.80	2.64	3.34	2.02	2.02	3.97	1.98
*SD*	.59	.50	.74	.54	.92	.86	.87	.83	.74	.74	.72	.64	.58	.64	.60

Note. OD = Optimistic disposition; SO = Sociability; EU = Emotional understanding; SE = Self-efficacy; AF = Affection; VA = Verbal aggressiveness; IM = Impression manipulativeness; QU = Questioning; EX = Expressiveness; EM = Emotionality; PR = Preciseness; PI = Personal impact; JD = Job dissatisfaction; SC = Social climate; QM = Quitting motivation. ***p < .05, ***p < .01,***p < .001.

With regard to the interaction of the variables with the Burnout components, Personal impact was found to be negatively correlated with all the Humanization factors, while Social climate was related positively in all cases. Job dissatisfaction was negatively related to Optimistic disposition, Sociability, Self-efficacy and Affection. Quitting motivation correlated negatively with Sociability and Affection.

Finally, the relationships between Communication and burnout emphasized positive correlations of Verbal aggressiveness, Impression manipulativeness, Questioningness and Emotionality repertoires with Burnout dimensions such as Personal impact, Personal dissatisfaction or Quitting Motivation. Preciseness in communications correlated positively with the Social climate dimension of Burnout.

### Humanization profiles: Comparative analysis of communication and burnout

**Fig 1:**
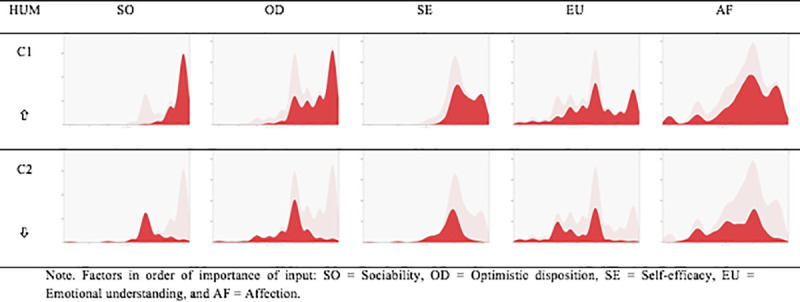
Cluster composition.

After confirming the relationship between the study variables, a two-stage cluster analysis was performed classifying cases by automatic distribution into clusters based on the mean scores on each of the Humanization dimensions (Fig 1).

The first (C1), made up of 63.3% of the cases (n = 209), was characterized by scores above the sample mean in all the Humanization dimensions: Sociability (M = 4.90), Optimistic disposition (M = 4.62), Self-efficacy (M = 4.41), Emotional understanding (M = 4.04), and Affection (M = 3.77). The second (C2), with 36.7% of the cases (n = 121), contrary to Cluster 1, assembled cases with scores below the sample mean in Sociability (M = 4.10), Optimistic disposition (M = 3.89), Self-efficacy (M = 3.78), Emotional understanding (M = 3.52), and Affection (M = 3.38).

After group classification into two clusters, a Student’s t test for independent samples was done to find out whether there were any differences between clusters with respect to the communication repertoires and with the dimensions of Burnout (Table 2).

**Table 2:** Communication and burnout. Descriptive statistics and *t* test by humanization cluster.

		HUM						*t*	*p*	*d*
		C1			C2					
		*N*	*M*	*SD*	*N*	*M*	*SD*			
Communication (CSI-R)	VA	209	1.78	.80	121	2.27	.87	-5.20***	.000	.60
	IM	209	1.65	.83	121	1.93	.91	-2.84**	.005	.33
	QU	209	2.21	.86	121	2.45	.76	-2.48*	.013	.28
	EX	209	2.79	.79	121	2.83	.65	-0.42	.675	-
	EM	209	2.56	.75	121	2.77	.70	-2.43*	.016	.28
	PR	209	3.43	.73	121	3.20	.68	2.80**	.005	.32
Burnout (CBB-R)	PI	209	1.88	.63	121	2.26	.60	-5.45***	.000	.62
	JD	209	1.95	.56	121	2.16	.59	-3.02**	.003	.35
	SC	209	4.12	.57	121	3.70	.68	5.99***	.000	.69
	QM	209	1.92	.62	121	2.13	.55	-2.14*	.033	.25

Note. VA = Verbal aggressiveness; IM = Impression manipulability; QU = Questioningness; EX = Expressiveness; EM = Emotionality; PR = Preciseness; PI = Personal impact; JD = Job dissatisfaction; SC = Social climate; QM = Quitting motivation. ***p < .05, ***p < .01, ***p < .001.

In the first place, communication scores were significantly higher in Cluster 2 in Verbal Aggressiveness, Impression manipulativeness, Questioningness and Emotion. Cluster 1 had a significantly higher mean score in Preciseness. Cluster 2 had significantly higher mean scores in the Personal impact, Job dissatisfaction and Quitting motivation dimensions of Burnout. However, the differences in Social climate were oriented in favor of Cluster 1.

### Communication styles as predictors of burnout in nursing

According to the data in Table 3, the regression analysis for each of the Burnout dimensions found an explained variance of 13.6% for Personal impact, 12.2% for Job dissatisfaction, 14.6% for Social climate and 20.9% for Quitting motivation. The Communication factors included in each of the models showed that Verbal aggressiveness was present in all of the Burnout dimensions, and was the only factor in Personal impact and also had the most weight in Personal dissatisfaction and Social climate. Preciseness was included as an explanatory variable in the model for Social climate. Meanwhile, Impression manipulativeness was one of the factors included in the Quitting motivation model.

**Table 3:** Stepwise multiple linear regression model for burnout dimensions.

Personal impact	Model	*R*	*R*^*2*^	*Corrected R*^*2*^	Change statistics					Durbin Watson
					Standard error of estimation	Change in *R*^*2*^	Change in *F*		Sig. of change in *F*	
	1	.36	.13	.13	.60	.13	51.57		.000	1.83
	Model 1		Unstandardized coefficients		Standardized coefficients		*t*	Sig.	Collinearity	
			*B*	Std. error.	Beta				Tol.	VIF
	(Constant)		1.47	.08			17.95	.000		
	Verbal aggressiveness		.27	.03	.36		7.18	.000	1.00	1.00
Job dissatisfaction	Model	*R*	*R*^*2*^	*Corrected R*^*2*^	Change statistics					Durbin Watson
					Change in *R*^*2*^	Change in *F*	Sig. of change in *F*		Change in *R*^*2*^	
	1	.32	.10	.10	.55	.10	35.13		.000	1.75
	2	.34	.12	.11	.54	.01	5.80		.017	
	Model 2		Unstandardized coefficients		Standardized coefficients		*t*	Sig.	Collinearity	
			*B*	Std. error.	Beta				Tol.	VIF
	(Constant)		1.47	.09			15.71	.000		
	Verbal aggressiveness		.14	.04	.21		3.03	.003	.58	1.69
	Questioning		.11	.04	.17		2.41	.017	.58	1.69
Social climate	Model	*R*	*R*^*2*^	*Corrected R*^*2*^	Change statistics					Durbin Watson
					Change in *R*^*2*^	Change in *F*	Sig. of change in *F*		Change in *R*^*2*^	
	1	.29	.08	.08	.62	.08	30.31		.000	1.75
	2	.38	.14	.14	.60	.06	23.56		.000	
	Model 2		Unstandardized coefficients		Standardized coefficients		*t*	Sig.	Collinearity	
			*B*	Std. error.	Beta				Tol.	VIF
	(Constant)		3.69	.16			22.43	.000		
	Verbal aggressiveness		-.24	.03	-.32		-6.32	.000	.98	1.02
	Preciseness		.22	.04	.25		4.85	.000	.98	1.02
Quitting motivation	Model	*R*	*R*^*2*^	*Corrected R*^*2*^	Change statistics					Durbin Watson
					Change in *R*^*2*^	Change in *F*	Sig. of change in *F*		Change in *R*^*2*^	
	1	.40	.16	.16	.55	.16	39.65		.000	1.67
	2	.43	.19	.18	.55	.02	6.14		.014	
	3	.45	.20	.19	.54	.01	4.75		.030	
	Model 3		Unstandardized coefficients		Standardized coefficients		*t*	Sig.	Collinearity	
			*B*	Std. error.	Beta				Tol.	VIF
	(Constant)		1.30	.11			11.45	.000		
	Questioningness		.23	.05	.33		4.03	.000	.59	1.68
	Verbal aggressiveness		.18	.06	.26		3.13	.002	.54	1.82
	Impression manip.		-.12	.05	-.17		-2.18	.030	.64	1.54

Finally, to check whether the relationships estimated were affected by multicollinearity, the tolerance of the variables and VIF (Variance inflation factor) were calculated. The results showed that absence of collinearity of variables included in the model may be assumed. The condition indices (PI: 4.74; JD: 7.85; SC: 11.30; QM: 8.41), did not reach the limit set by Belsley [43].

### Communication mediation on the relationship between humanization and burnout

In answer to the mediation hypothesis, whether the presence of certain communication repertoires mediates in the relationship between Humanization and Burnout levels, simple mediation analyses were performed. In all cases, the predictor variables pertaining to a cluster based on Humanization scores (X), the Burnout dimensions (Y) and communication styles (M_1_, M_2_,…), were entered as mediator variable in each of the models (depending on results from previous regression analyses).

**Fig 2:**
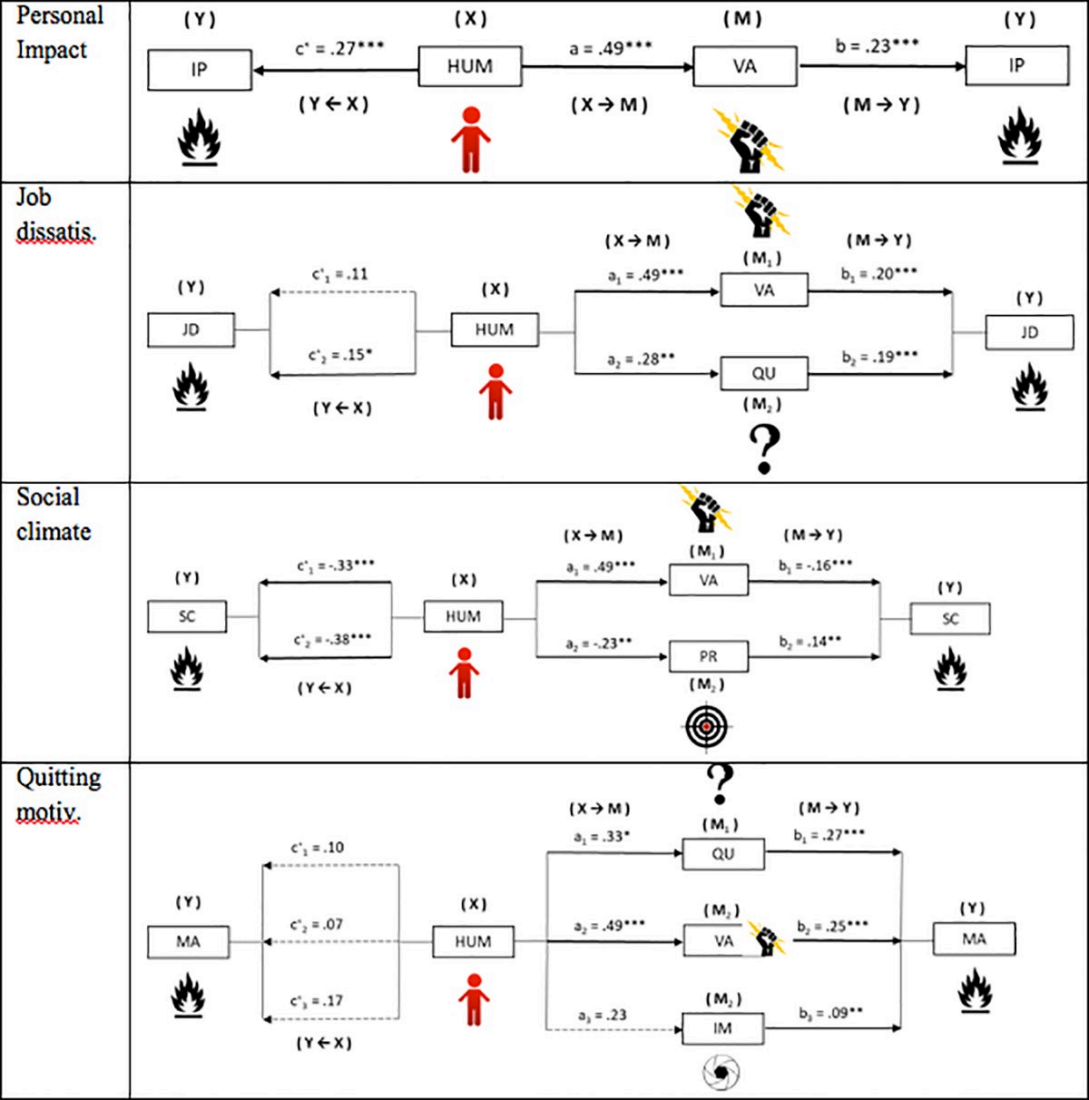
Mediation model of communication styles on the relationship between humanization and personal impact, job dissatisfaction, social climate and quitting motivation.

Fig 2 shows the results of the simple mediation models for the Personal impact dimension of Burnout. Significant effects of Humanization were observed on Verbal aggressiveness (B = .49, p < .001), and on the Personal impact component of burnout (B = .23, p < .001). The model shows a direct effect of Humanization on Personal impact of B = .27, p < .001. Significant values were found [B = .11, SE = .03, 95% CI (.065, .187)] with the analysis of indirect effects (X→M→Y) by *bootstrapping*.

The simple mediation models for Job dissatisfaction show significant effects of Humanization on the Verbal aggressiveness (B = .49, p < .001) and Questioningness (B = .28, p < .01) mediators. The M→Y effects were significant in both cases (VA: B = .20, p < .001; QU: B = .19, p < .001), with a total effect of the model of B = .21, p < .01. The analysis of indirect effects revealed significance of both Verbal aggressiveness [B = .09, SE = .03, 95% CI (.049, .172)], and Questioningness [B = .05, SE = .02, 95% CI (.020, .110)].

First, for the Social climate dimension of Burnout, significant effects of Humanization (X) were observed on the Communication factors included as mediators: VA [B = .49, p < .001], and PR [B = -.23, p < .01]. M→Y effects estimated were found to be significant in both cases: VA [B = -.16, p < .001], and PR [B = .14, p < .01]. Finally, the analysis of indirect effects found significant values in the two models computed: VA [B = -.08, SE = .02, 95% CI (-.140, -.041)], and PR [B = -.03, SE = .01, 95% CI (-.083, -.007)].

The results of the mediation analysis for Quitting motivation showed that the direct effects of Humanization were not significant. The effect on communication styles as mediator variables was significant for Questioningness (B = .33, p < .05) and Verbal aggressiveness (Y), demonstrating the significance of QU (B = .27, p < .001), VA (B = .25, p < .001), and IM [B = .09, p < .01].

Finally, the analysis of indirect effects found significant values in two of the assumptions: QU [B = .09, SE = .03, 95% CI (.030, .180)], and VA [B = .12, SE = .04, 95% CI (.060, .220)]. On the contrary, for the model in which impression manipulativeness was included as a mediator, the indirect effects were not significant [B = .02, SE = .02, 95% CI (-.001, .084)].

## Discussion

In the first place, with respect to the relationship between the dimensions of humanization and communication styles, our results showed the close negative association between the HUMAS dimensions related to affection and a communication style based on verbal aggressiveness, questioningness and emotionality. Therefore, when nurses had low scores on affection, they were associated above all with high scores in verbal aggressiveness, questioningness and emotionality. Thus, it would have to be determined where the limit in affection should be set for it not to be associated with an aggressive, questioning communication style loaded with stress and worry, as according to previous studies, excessive affective empathy could also cause burnout in nurses [2, 4, 33]. The Affection dimension moderately correlated positively with a communication style characterized by impression manipulativeness, where the effect size was also medium. Since self-efficacy is related to less perceived stress, this association could be contributing to better communication, although the role of the personality variables, such as neuroticism, in that association should be borne in mind [1, 3, 4, 36].

Of the associations between the dimensions of humanization and the components of Burnout, the negative correlation between the affection dimension of humanization and Social impact was highly significant, as was the positive relationship of optimism with the HUMAS self-efficacy and social climate dimensions. Nurses with the highest scores in affection scored lower in impact of burnout in different vital areas, so in this case, affection favored wellbeing, but as mentioned above, more in-depth studies are needed to find out what the healthiest level of affection is and its relationship with other variables, such as emotional intelligence [44].

Moreover, the higher the scores in optimism and self-efficacy, the better social climate was. Some studies have found that self-efficacy was related to self-esteem and integration in the setting [1] and optimism has been associated with work commitment [8], supporting the results above. Other negative and positive correlations with a moderate effect size were found. Among the negative correlations were the association between the optimistic disposition and self-efficacy humanization dimensions and the personal impact component of burnout, as well as affection in humanization, job dissatisfaction and quitting motivation associated with the burnout syndrome. The association of sociability and emotional understanding in humanization and the association of social climate with burnout were positive.

The communication and burnout correlations were highly positive in communication styles based on verbal aggressiveness and questioningness in the care relationship and quitting motivation which are involved in the burnout syndrome. Considering the similar findings of other researchers suggesting the relevance of intrinsic and extrinsic motivation against burnout [33], we think that increasing motivation might improve communication styles.

Second, different humanization profiles were found in the nurses. The cluster analysis identified a group (63.3% of the sample) which scored above the mean in optimistic disposition, sociability, emotional understanding, self-efficacy and affection, and another group (36.7% of the sample) which scored below the mean in the HUMAS dimensions mentioned. Researchers agree that humanization is related with more effective and assertive communication [10, 22–24] and recent studies have revealed that burnout impedes humanization in care [3, 32]. Therefore, as over 30% had lower levels in humanization, it would be advisable to improve humanization and communication in nurses to reduce burnout [31, 44] for a review of humanization in care.

Third, the objective of developing a predictive model for each of the burnout components based on communication styles was met. The results were relevant for each of the components of burnout as described under Results. It was observed a moderate predictive power of communication styles in the quitting motivation component of burnout. The model explained 20.9% of the variability in quitting motivation of nurses, so quitting motivation increased when the communication style was characterized by verbal aggression, impression manipulativeness and especially, by questioningness. That is, the lack of personal promotion and growth in one’s work was greater when inappropriate communication styles predominated. This result shows clear implications for professional practice because an adequate communication style could increase satisfaction and stress reduction, furthermore help to improve adherence to treatment and reduce the stress of patients too.

Although verbal aggressiveness explained the four components of burnout (personal impact, social climate, job dissatisfaction and quitting motivation), it had the most explanatory weight in social climate and job dissatisfaction associated with burnout. Verbal aggressiveness positively influenced personal impact, explaining 13.6% of the variability in burnout.

Fourth, the objective of computing mediation models based on the indirect influence of communication styles on the relationship between humanization profiles and the burnout components. Verbal aggressiveness influenced the effects that humanization had on burnout, increasing personal impact and job dissatisfaction associated burnout and decreasing social climate. A communication style based on questioningness also had an indirect effect on the relationship between humanization profiles and job dissatisfaction, in the same direction as verbal aggressiveness. These results are congruent with those of previous studies which support significant relationships between communication and job satisfaction [26–29].

Some limitations of this study should be mentioned. First the study was limited by the small sample size and also related to sample it is specific to the sphere of nursing and sampling method (snow ball), all this makes that we should be prudent in generalizing the results to other healthcare professionals and specializations. Second, related to the way data were collected, as the mean age was low with respect to the reality of the Spanish nurses, and their competences on the use of new technology tools with which the questionnaire was publicized and data were collected. Third, there exists a limitation derived from the study design, because, as a cross-sectional study there were variables which could not be controlled. So future studies could use other strategies for data collection, including a large population of nurses and other health professionals even.

## Conclusions

The first conclusion is that nursing personnel must be open to communication that is characterized by preciseness, through adequate interpretation of the thoughts, feelings and attitudes of the other person, with cognitive empathy, to be able to offer humanized care. Finally, ineffective communication styles intervene indirectly in the relationship between humanized care (diminishes) and burnout (increases).

Based on these findings, it is recommended that nurses be trained in communication styles and development of personal competencies which humanize their professional performance, cognitive empathy and preciseness in communication, and within the framework of patient-centered care, with an eminently practical focus on the profession, where humanized care and problem-solving are learned in the clinical environment. We also emphasize the importance of professions whose competency in humanization is adequate, strengthening commitment to care. These competencies should reinforce preciseness in communication, and therefore, improve the climate of personal and professional relations in the workplace.

The development and implementation of training programs to promote personal competencies related communication skills, emotional competencies or initiatives that have proven effective in reducing mild mental disturbances in situations with a high emotional impact, like clinical practice, such as practice mindfulness or interventions focused in improving soft skills.

## Supporting information

S1 File (SAV)
